# Supplementary material for: Splice-Junction-Based Mapping of Alternative Isoforms in the Human Proteome
Source: Cell Rep. Author manuscript; Available in PMC 2020 Jan 15. (PMC6961840; doi:10.1016/j.celrep.2019.11.026)
Supplement: 2 [file NIHMS1546469-supplement-2.zip › DF1/PXD006675/RightAtrium/RightAtrium_7_TMEM245_ESSEAVDRGESAPTLSTSPSPSSPSPTSPSPTLGR.pdf]

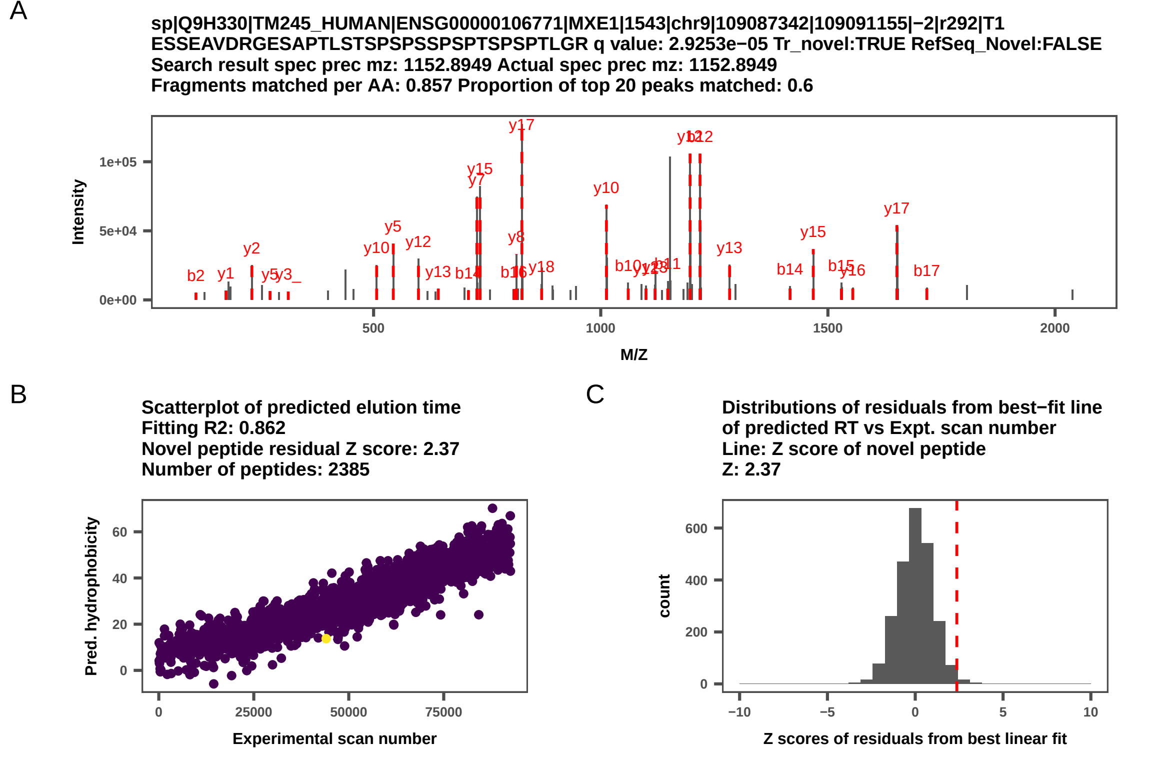

A
sp|Q9H330|TM245_HUMAN|ENSG00000106771|MXE1|1543|chr9|109087342|109091155|−2|r292|T1
ESSEAVDRGESAPTLSTSPSPSSPSPTSPSPTLGR q value: 2.9253e−05 Tr_novel:TRUE RefSeq_Novel:FALSE
Search result spec prec mz: 1152.8949 Actual spec prec mz: 1152.8949
Fragments matched per AA: 0.857 Proportion of top 20 peaks matched: 0.6
Intensity
1e+05
5e+04
0e+00
M/Z
500
1000
1500
2000
B
Scatterplot of predicted elution time
Fitting R2: 0.862
Novel peptide residual Z score: 2.37
Number of peptides: 2385
Pred. hydrophobicity
Experimental scan number
C
Distributions of residuals from best−fit line
of predicted RT vs Expt. scan number
Line: Z score of novel peptide
Z: 2.37
count
Z scores of residuals from best linear fit
